# Multivariate profiling of neurodegeneration-associated changes in a subcellular compartment of neurons via image processing

**DOI:** 10.1186/1756-0381-1-10

**Published:** 2008-11-14

**Authors:** Saravana K Kumarasamy, Yunshi Wang, Vignesh Viswanathan, Rachel S Kraut

**Affiliations:** 1 Institute of Bioengineering and Nanotechnology, 31 Biopolis Way, the Nanos, #04-01, 138669, Singapore

## Abstract

**Background:**

Dysfunction in the endolysosome, a late endosomal to lysosomal degradative intracellular compartment, is an early hallmark of some neurodegenerative diseases, in particular Alzheimer's disease. However, the subtle morphological changes in compartments of affected neurons are difficult to quantify quickly and reliably, making this phenotype inaccessible as either an early diagnostic marker, or as a read-out for drug screening.

**Methods:**

We present a method for automatic detection of fluorescently labeled endolysosomes in degenerative neurons in situ. The Drosophila *blue cheese *(*bchs*) mutant was taken as a genetic neurodegenerative model for direct in situ visualization and quantification of endolysosomal compartments in affected neurons. Endolysosomal compartments were first detected automatically from 2-D image sections using a combination of point-wise multi-scale correlation and normalized correlation operations. This detection algorithm performed well at recognizing fluorescent endolysosomes, unlike conventional convolution methods, which are confounded by variable intensity levels and background noise. Morphological feature differences between endolysosomes from wild type vs. degenerative neurons were then quantified by multivariate profiling and support vector machine (SVM) classification based on compartment density, size and contrast distribution. Finally, we ranked these distributions according to their profiling accuracy, based on the backward elimination method.

**Results:**

This analysis revealed a statistically significant difference between the neurodegenerative phenotype and the wild type up to a 99.9% confidence interval. Differences between the wild type and phenotypes resulting from overexpression of the Bchs protein are detectable by contrast variations, whereas both size and contrast variations distinguish the wild type from either of the loss of function alleles *bchs1 *or *bchs58*. In contrast, the density measurement differentiates all three *bchs *phenotypes (loss of function as well as overexpression) from the wild type.

**Conclusion:**

Our model demonstrates that neurodegeneration-associated endolysosomal defects can be detected, analyzed, and classified rapidly and accurately as a diagnostic imaging-based screening tool.

## Background

Major efforts are underway to identify drug candidates for the treatment of Alzheimer's disease. Most of these are aimed at interference with the production or activity of the amyloid peptide Aβ, since this is the most likely causative agent of the disease [1]. However, other cell biological phenomena such as degradative trafficking to the lysosome have been identified as playing an important role in the disease progression [2].

The endolysosome refers to a vesicular degradative organelle spanning the late endosomal and lysosomal compartments [3]. Endolysosomal compartments are found in neuronal cell bodies, and are transported along axons to and from synaptic termini in both mammals and flies [4-6]. The involvement of the endolysosomal system in neurodegeneration is suggested by defects in the morphology, enzymatic sorting, and function of these compartments which accompany early stages of diseases such as Alzheimer's [7,8]. In fact, Cataldo et al. [9] have shown that there are morphometric differences in the endolysosomal compartments of neurons in the central nervous system of normal and Alzheimer's disease afflicted human subjects. Large-scale and unbiased assessment of morphological abnormalities in the endolysosomal compartment of degenerating neurons either in vivo or in cell culture would be valuable both to define a recognizable neurodegenerative state, and to provide a readout for high-throughput cell-based screening paradigms.

Neuronal loss- and gain-of-function in *blue cheese *(*bchs*), a putative lysosomal transport protein in Drosophila [10,11] lead to a phenotype that displays features associated with human neurodegenerative diseases. These include neuronal death, ubiquitin-rich brain inclusions, and shortened lifespan in adults [10] and disintegration of axonal processes, cytoskeletal defects, slowed axonal transport and motorneuronal death in third instar larvae (Lim and Kraut, manuscript under revision). Here, we describe alterations in endolysosomal size and density accompanying the neurodegenerative phenotype in motorneurons of *blue cheese *mutant larvae. This system was chosen for examining neurodegenerative endolysosomal changes because of several advantages over other neurodegeneration models. First, the neurodegenerative phenotype in *blue cheese *comes about due to a defect in a presumptive lysosomal processing pathway [11], as opposed to other commonly used mouse and fly models, which rely on overexpression of a human gene [10,12,13]. Secondly the existence of identified motorneurons in which degeneration takes place makes this model unique (Lim & Kraut, submitted). Both of these features remove ambiguity in interpreting the data. Additionally, the ease of preparation of the neurodegenerative mutant animals, and the fact that motorneurons in the fly larva are close to the surface, provide a practical advantage in collecting large numbers of high-quality images.

Previously, changes in endolysosomal morphology and/or number have been noted under various neurodegenerative conditions [8,9]. However, these were described in a labor-intensive and not easily quantifiable manner, and therefore did not yield parameters that could be applied to rapid analysis of either in vivo animal models or drug screening platforms. Since manual quantification of endolysosomal features is highly subjective and time consuming, a rapid and efficient method of assessing these features would be desirable as the basis for a novel screening paradigm. Loo et al. [14] have implemented a multivariate drug screening approach on human cancer cells, where the phenotypic effects of various drugs were classified based on 300 feature measurements. Here, we test the efficacy of using *in situ *profiling of neuronal endolysosomes as a potential platform for morphogenetic screening. In our method, the endolysosomes are visualized by transgenic expression in isolated motorneurons of a green fluorescent protein (GFP)-fusion of an endolysosomal protein, Spinster[15]. This marker has the advantage that it can be expressed in isolated neurons of choice using the Gal4-UAS system [16] and that it is endolysosomally localized. Fluorescently labeled compartments appear as fluorescent spots in 2-D image sections of neuron termini, which synapse onto muscles at the neuromuscular junction (NMJ)[17].

The analysis presented here first uses a novel image filtering method to detect fluorescently labeled endolysosomal compartments, which appear as spots, in the NMJs of larval motorneurons. The detection method relies in its ability to segment images of neurons robustly and accurately in situ to yield endolysosomal compartments, or spots, as regions of interest (ROIs) that can be analyzed objectively. Standard segmentation methods based on edge detection [18] and intensity thresholding [19] performed poorly in recognizing spots in our images, since some spots have very weak edges and/or intensities and therefore fall below the detection threshold. The presence of background fluorescence or noise also exacerbated the problem of accurately detecting spots. Here, we are able to detect both strong and weak spots while suppressing background noise by identifying regions in the image with a high point-wise multi-scale correlation and normalized cross-correlation [20,21] to that of a Laplacian of Gaussian (LoG) filter [22,23].

The second step of our method uses a support vector machine (SVM) classifier [24] with a radial basis function to determine the differences in the contrast, size and density features between the wild type and the neurodegenerative image sets. We also rank the features, based on their profiling accuracy, via the backward elimination scheme [25]. Univariate and multivariate analyses were used to rank various features of the endolysosomal compartments (e.g. size, density, intensity) in terms of discriminating power in order to assess the importance of each feature in determining the ability of the SVM to predict genotypic origin. This identified the feature or features that could be used most efficiently to assign a class, either neurodegenerative or WT, to a given sample, and also allowed the visualization of differences between each wild type vs. mutant pair. Finally, Fisher's Linear Discriminant analysis (FLD) [26,27] was used to visualize the underlying differences between the wild type and the various mutant classes.

The novelty of this study lies in the application of standard machine learning and statistical analytical methods to an in-vivo neurodegenerative model. It has previously been noted that the endolysosomal compartments of degenerating neurons are affected not only functionally, but also in terms of appearance, e.g. size, shape, and density [9,28]. Our study now shows that rapid *in situ *profiling of morphological features of endolysosomal compartments is possible, and could therefore serve as a potential platform for morphogenetic screening of neurodegenerative diseases either in animal or cell culture models. The method consists of imaging a large number of motorneuronal endolysosomal compartments of diverse contrast levels, whose features are not visually obvious, and are not accessible by conventional measurement methods. The SVM classifier evaluates changes in contrast, size and density features of these compartments in a rapid, unbiased manner, and on a far larger quantity of image data than would be possible manually, e.g. by analysis of electron micrographs, or fluorescence images.

To our knowledge, this is the first reported image-based multivariate analysis of a neurodegeneration-related feature at the cell biological level. The strength of the approach is that it can identify and quantitatively describe a diagnostic aspect of the neurodegenerative phenotype, namely alterations in key endolysosomal features. Because of its speed, robustness, and accuracy, the proposed method has potential applications in screening the palliative effects of anti-neurodegenerative drugs on an in vivo model.

## Methods

### Preparation of Larvae for Imaging

Third instar Drosophila larvae carrying a transgene for the green fluorescent fusion protein spinster-GFP[15] in wild type or in a genetic background mutant in the *blue cheese *gene [10] or in a background carrying an overexpression construct in *blue cheese *EP2299 (Enhancer-Promoter line 2299) [29,30], were analyzed. Expression of the endolysosomal marker spinster-GFP was driven in two identified motorneurons, which are referred to using the abbreviations aCC and RP2 (see reference [17]) of each hemisegment using the GAL4-UAS system [16], with an *even skipped *driver line (designated RRa > spinster-GFP, where the symbol ">" refers to a GAL4 line driving a reporter. RRa was the abbreviated name given to the *even skipped *driver line described in [31]). Genotypes were as follows:

i. $\frac{+}{+};\frac{RRa>Spinster-GFP}{RRa}$

ii. $\frac{bchs1}{Df(2L)c\text{l}7};\frac{RRa>Spinster-GFP}{RRa}$

iii. $\frac{bchs58}{Df(2L)c17};\frac{RRa>Spinster-GFP}{RRa}$

iv. $\frac{EP2299}{+};\frac{RRa>Spinster-GFP}{RRa}$

The aforementioned genotypes will henceforth be referred to as i. Wild type ii. *bchs1 *iii. *bchs58 *and iv. EP+ respectively. *Df(2L)c17 *and *bchs1 *are genetic deletions and strong loss of function alleles of *blue cheese *[10] while *bchs58 *carries a stop mutation in the *blue cheese *coding region, but does not produce detectable protein (own observations and [32]). Larval fillets were prepared, immunostained for GFP, and mounted as described previously [30]. Neuromuscular junctions (NMJs) of the aCC motorneuron in abdominal hemisegments number 2–7 (A2-A7; 12 hemisegments per animal) were imaged and NMJs for all animals of a given genotype were pooled.

### Image Acquisition

The images were acquired with a 60×/1.42NA lens at 490 nm excitation/528 nm emission wavelengths using the Deltavision Fluorescence deconvolution imaging system (SoftWorx; Applied Precision, Seattle) equipped with a motorized Olympus IX70 inverted wide-field fluorescence microscope, a 12 bit Coolsnap HQ CCD camera and a 100 W mercury lamp. A stack of 512 × 1024 pixel optical sections spaced at 0.3 μm was acquired for every NMJ in the larvae samples with no binning. The sampling pixel size was 0.1 μm × 0.1 μm along the *x*- and *y*- axes which provided sufficient resolution for imaging the endolysosomal compartments.

Three morphological parameters were used to characterize the differences between endolysosomal compartments in wild type and in each of the different mutant genotypes as shown in Table 1. The exposure time and illumination intensity were fixed for each pair-wise comparative study (i.e. WT vs. a particular *blue cheese *allele) since we determined that certain measurements were sensitive to variations in exposure conditions (not shown). However, it was not possible to use a common acquisition setting for all three studies due to the widely varying fluorescence of the spots between the different groups. The acquisition setting was selected such that the dynamic range of the CCD chip was not exceeded, and an optimal contrast of the endolysosomal compartments with respect to the background was achieved. Images were deconvolved using the constrained iterative algorithm [33] provided with SoftWorx.

**Table 1:** Image acquisition settings for wild type vs. EP+, bchs1 and bchs58

Study	Exposure time (ms)	Illumination intensity (% transmission)
Wild type vs. EP+	0.5	10
Wild type vs. *bchs1*	0.3	10
Wild type vs. *bchs*58	0.3	10

### Image Processing

Endolysosomal compartments within NMJs were imaged in 2-D optical sections and their contrast, size and density were quantified via the following three sequential steps: (i) enhancement of NMJ spots, (ii) detection and labelling of NMJ spots, and (iii) extraction of spot size, density and contrast.

#### Enhancement of NMJ spots

The normalized cross-correlation (NCC) as a measure of the similarity between the local spot intensity profile and a predefined Laplacian of Gaussian (LoG) filter was calculated as follows:

(1) $$\text{NCC}\left(x\text{,}y\right)=\frac{\langle b_{x,y}-{\bar{b}}_{x,y},g_{7\times 7}-\bar{g}\rangle }{\left\|b_{x,y}-{\bar{b}}_{x,y}\right\|\left\|g_{7\times 7}-\bar{g}\right\|}$$

where **b**_*x*,*y *_and **g**_7 × 7 _are column-wise vector representations of the local image neighbourhood centred at pixel coordinates (*x*, *y*) and the 7 × 7 LoG spot filter respectively whereas ${\bar{b}}_{x,y}$ and $\bar{g}$ are the mean values of **b**_*x*, *y *_and **g**_7 × 7 _respectively.

The point-wise multi-scale correlation (MSC) is given by eqn. (2). The MSC measure is based on the degree of correlation of a localized region at (*x*, *y*) to a total of *J *"spot-like" LoG filters

(2) $$\text{MSC}\left(x,y\right)=\sqrt[{J}]{\prod_{i=1}^{J}C_{i}\left(x,y\right)}$$

where *C*_*i *_is a correlation measure formally expressed as *C*_*i*_(*x*, *y*) = ⟨**b**_*x*,*y*_, **g**_2*i*+1 × 2*i*+ 1_⟩, 2*i*+1 denotes the filter dimension where *i *= 1, 2, ..., *J *and *J *= 5 in this case.

#### Detection and Labeling of NMJ Spots

Combined NCC and MSC detection was done by first detecting regions whose normalized cross correlation magnitude exceeded a predefined threshold *T*_1 _i.e. |NCC(*x*, *y*)| > *T*_1_.

Spots detected by the NCC were also filtered to assure that the magnitudes of the point-wise MSC measures exceeded another predefined threshold *T*_2 _i.e. |MSC(*x*, *y*)| > *T*_2_. A sufficiently low threshold value was determined for *T*_2_, via a trial and error approach on the available set of image sections, such that the spot regions were effectively distinguished from noise. Appropriate *T*_1 _and *T*_2 _levels (*T*_1 _= 0.4 and *T*_2 _= 23) were chosen from a range of threshold *T*_1 _and *T*_2 _values that were empirically tested for their classification accuracy (see fig. 5). These threshold values were used for the measurements of all genotypes. After detection, a unique label was assigned to each spot using 2-D connected components labelling [34] so that the contrast and size information of each spot could then be extracted.

**Figure 1:**
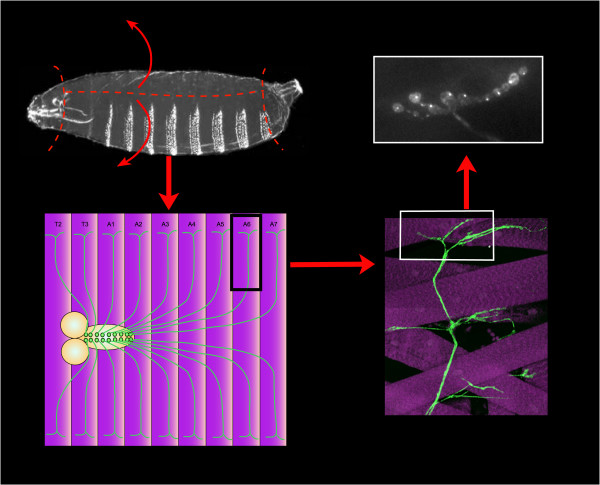
**Topographic representation of R-SVM classification accuracy over a range of threshold values for spot detection**. Normalized classification accuracy outcome after empirical measurement using a range of values for the NCC threshold *T*_1 _and the MSC threshold T_2_, where Δ*T*_1 _= 0.1 and Δ*T*_2 _= 5, with 1 (white areas) representing 100% accuracy, and 0.93 (black areas) representing 93% accuracy. Threshold values of *T*_1 _= 0.4 and *T*_2 _= 23 were used in the actual measurements.

#### Extraction of Size, Density and Contrast

For feature measurements, size information was encoded as a histogram distribution with 10 size categories (*S*_1_, *S*_2_, ..., *S*_10_) ranging from 0.01 μm^2 ^to 0.1 μm^2 ^(1–10 pixels). Contrast information was also encoded as a histogram distribution with 14 categories (*C*_1_, *C*_2_, ..., *C*_14_) uniformly divided within the 0–255 gray-scale where the contrast measurement of a spot is defined as the difference between the peak intensity of the spot and the background intensity surrounding it. The background intensity follows the minimum pixel value within a 15 × 15 window centered around the peak intensity location. A 15 × 15 window size was chosen since it is sufficiently large to contain any given spot.

The feature "spot density" was obtained by determining the centroid of each spot, corresponding to the location of its peak intensity. Subsequently a circular area with a fixed radius *r *was defined around each spot so that the spot density could be translated as the degree of overlap between these circles. The radius *r *was selected such that the circles were larger than the spots, and the diameter (2*r*) typically resembled the average distance between adjacent spots for the wild type case. We formally define spot density *D *as

(3) $$D=\frac{N\pi r^{2}-A}{A\left(N-1\right)}$$

where *N *is the number of spots, *r *is the fixed radius and *A *is the total area occupied by the spots. *D *is normalized such that it lies between 0 and 1. Smaller areas of overlap give a lower spot density. The accuracy of the density measure is adversely affected if there are too few spots or if the spot distribution in an NMJ section is nonuniform. To ensure its robustness, we compute the density measure for cases where the spot population is sufficiently large (*N *≥ 50). We observe that the spot distribution in the NMJs is generally uniform. However, in any given NMJ section, there may be two or more clusters of spots that are disjoint due to the intervening regions being out of focus at that focal section. In such cases, the density measure is based on the largest cluster of spots in that section.

### SVM Classification

Acquired feature data was analyzed using a radial basis function Support Vector Machine (R-SVM) classifier [24]. A leave-one-out cross validation technique was applied to determine the classification accuracy of a dedicated R-SVM for each pair of classes. The data set in each pair was normalized such that each entry had a zero mean and unit standard deviation. Since the R-SVM requires the penalty factor (*φ*) and kernel width of the radial function (*σ*) as input arguments, we selected *φ' *and *σ' *which gave the highest classification accuracy from the following range of input values *φ *= {0.1, 1, 10, 100, 1000} and 0.2 ≤ *σ *≤ 40 where the step size Δ*σ *= 0.2. Once implemented, R-SVM(*φ'*, *σ'*) outputs a decision value *d*_*i *_for every input feature vector **x**_*i *_where the sign of *d*_*i *_is used to predict the class to which **x**_*i *_belongs and the magnitude is a measure of the distance of **x**_*i *_from the decision boundary.

### Statistical Data Analysis

#### Feature Ranking – Univariate Analysis

The discriminating power of individual features was quantified based on the Student's t-test [27] value where discriminability is measured by the magnitude of the *t*^2 ^value which is expressed as follows

(4) $$t=\frac{{\left(m_{1}-m_{2}\right)}^{2}}{\sigma_{1}/n_{1}+\sigma_{2}/n_{2}}$$

where *m*_1 _and *m*_2 _are the mean values of a given feature in the wild type and mutant class respectively, *σ*_1 _and *σ*_2 _are the corresponding standard deviation values and *n*_1 _and *n*_2 _are the sample sizes of the two classes.

#### Feature Ranking – Multivariate Analysis

The Hotelling t-test [27] in eqn. 5, which is the generalized form of the t-test in eqn.4, is given by:

(5) *T *= (**m**_1 _- **m**_2_)'**S**^-1^(**m**_1 _- **m**_2_)

The notations **m**_1 _and **m**_2 _denote the mean distribution of feature vectors in the two classes whereas **S **= **S**_1_/*n*_1 _+ **S**_2_/*n*_2 _given that **S**_1 _and **S**_2 _are the corresponding covariance matrices.

#### Data Visualization – Univariate Analysis

The differences between the wild type and mutant mean distribution profiles for contrast and size were plotted in histograms. The scalar value for spot density was plotted as a probability density function with a Gaussian distribution characterized by the mean and standard deviation in spot density values from a given genotype.

#### Data Visualization – Multivariate Analysis

Fisher's linear discriminant [26] is applied to visualize the endolysosomal differences between the wild type and mutants in 3-D feature space defined by the axes **ω**_12_, **ω**_13 _and **ω**_14 _which are projection vectors that optimally separate wild type vs. *bchs*1, wild type vs. *bchs*58 and wild type vs. EP+ respectively. They are formally defined as follows:

(6) $$w_{ij}=S_{ij}^{-1}\left(m_{i}-m_{j}\right)$$

## Results

### Detection of endolysosomal compartments in a neurodegenerative model

The Drosophila neurodegenerative mutant *bchs *was chosen as a model system in which to analyze changes in the morphology of endolysosomal compartments in degenerating neurons in situ. Neuronal degeneration in *bchs *mutant animals has been well-described and is believed to affect endolysosomal function and trafficking [10,11](Lim and Kraut, manuscript under revision). Drosophila strains were created that were mutant for *bchs *or overexpressing Bchs in motorneurons and that also expressed the endolysosomal marker spinster-GFP in motorneurons known to be affected by the neurodegenerative phenotype (A. Lim and R. Kraut, manuscript under revision). Third instar larvae of the relevant genetic backgrounds were filleted and neuromuscular termini at the body wall were imaged with high-resolution wide-field microscopy (see methods, and fig. 1).

**Figure 2:**
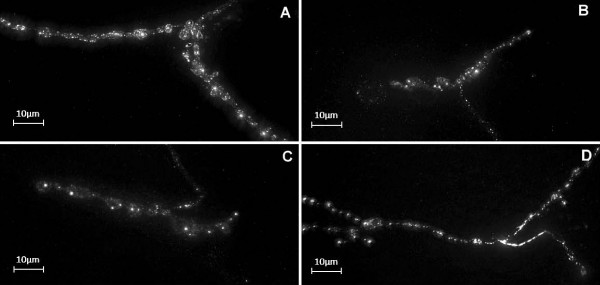
**Preparation of Drosophila larvae for imaging labelled endolysosomal compartments in nerve termini**. Third instar larvae are dissected and opened out as indicated (upper left) to reveal the brain and larval motorneurons with axons (green) extending into the peripheral muscle field, with segments labelled T2-A7 (purple; lower left). A bundle of axons, including the aCC and RP2 motoraxons (see text) and expressing membrane-localized EGFP is shown making branching contacts with dorsal muscle fibers (lower right) in an actual fixed and immunostained preparation of a larval body wall. The nerve terminal (or neuromuscular junction, NMJ) is boxed and the equivalent area of an animal expressing spinster-GFP is shown (upper right), highlighting the punctate endolysosomal compartments in the aCC terminal.

The structure of interest was the Drosophila larval neuromuscular junction (NMJ) (shown schematically in fig. 1). The NMJ refers to the structure at the terminus of the motorneuron that extends its axon into the peripheral muscle field in the body wall of the larva, and makes numerous synaptic contacts with muscle targets. Analysis of image stacks of labelled NMJs after filleting, immunofluorescent labelling of endolysosomal compartments, wide-field imaging, and deconvolution were carried out as follows:

A) Differences in endolysosomal compartment features, including spot contrast, size, and density of spots, were quantified and compared between the wild type and the three mutant classes using a radial basis support vector machine (R-SVM).

B) 25 contrast, size and spot density features were ranked in terms of their discriminating abilities using the backward elimination scheme [25].

C) The differences in features between the wild type and the mutants were visualized from both univariate feature analysis and Fisher's linear discriminant [26,27].

Fig. 2 shows a typical maximum projection view of an NMJ from the wild type and mutant genotypes, expressing the endolysosomal marker spinster-GFP in two motorneurons (aCC and RP2) terminating at the NMJ on dorsal muscles. There are fewer compartments per area in the *bchs *mutants (Fig. 2b and 2c) compared to the wild type although most *bchs1 *spots appear more prominent. The EP+ NMJ (Fig. 2d) has brighter spots than the wild type.

**Figure 3:**
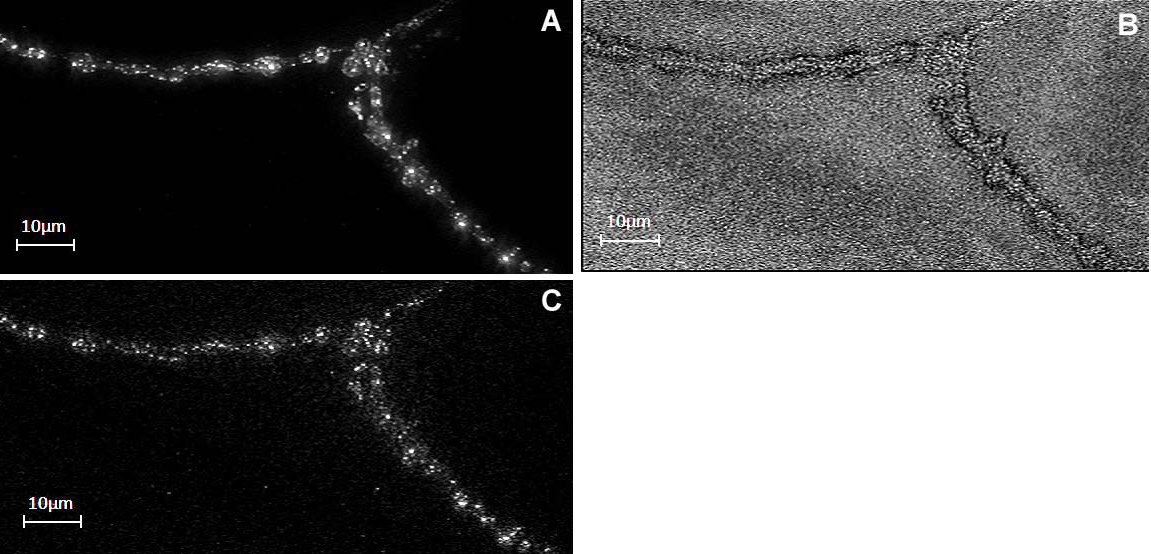
**Spinster-GFP tagged endolysosomal compartments in motorneuron termini**. Projected stacks of deconvolved images from NMJs of (A) Wild type (B) *bchs1 *(C) *bchs58 *and (D) EP+ genetic backgrounds, expressing spinster-GFP in endolysosomal compartments. The fluorescently labelled endolysosomal compartments appear as bright spots within the NMJ region of the motorneuron. For purposes of presentation in this figure, images have been adjusted for brightness and contrast.

In order to overcome the problem of differing intensities and contrast in fluorescently labelled endolysosomal compartments, which make detection by standard convolution algorithms difficult [35], we devised a spot enhancement scheme employing two correlation techniques: normalized cross correlation (NCC) [21] and point-wise multi-scale correlation (MSC). NCC has been widely used in frame averaging [20] and edge detection [36]. Here we used it to identify both bright and faint spots accurately based on the similarity of their local spot intensity profiles to a predefined Laplacian of Gaussian (LoG) filter. The normalized NCC measure thus resulted in accurate spot localization regardless of intensity, but was sensitive to background noise. Application of the MSC, on the other hand (see Methods, eqn. (2)) gave significantly better SNR but was sensitive to spot intensity, i.e. weaker spots were not always detected using MSC alone. However in combination with NCC, MSC was able to assign higher coefficient values to bona fide spots compared to background noise. Thus, spots were detected first by including regions whose NCC magnitude exceeded a predefined threshold *T*_1 _(see Methods, *detection and labelling of NMJ spots*), and potential artefacts or noise were eliminated by ensuring that the detected regions also had corresponding point-wise MSC measures whose magnitudes exceeded another predefined threshold *T*_2 _(see Methods, *detection and labelling of NMJ spots*).

**Figure 4:**
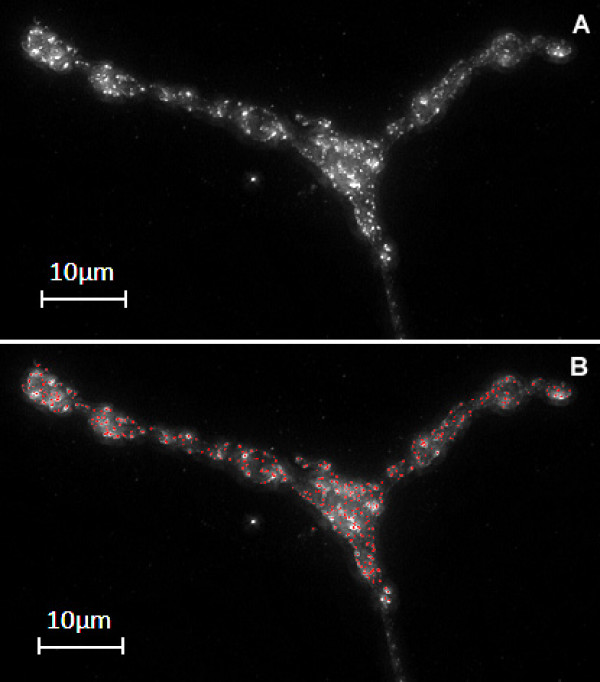
**NCC and MSC spot detection and enhancement filters produce different outputs**. (A) Sample 2-D image section of an NMJ. (B) the normalized cross correlation output, NCC, detects spots as well as background noise. (C) Point-wise multi-scale correlation measurement, MSC, suppresses background noise but emphasizes brighter spots such that weaker spots may fall below the detection threshold. For purposes of presentation in this figure, images in A and C have been adjusted for brightness and contrast.

**Figure 5:**
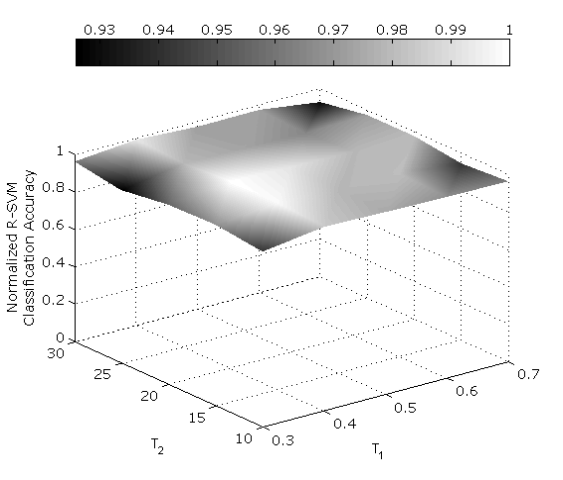
**Accurate detection of faint and strong spots while suppressing background noise**. Figure 4(A) A 2-D maximum projection from a deconvolved stack of a wild type NMJ. (B) Endolysosomal spots detected from the projection using NCC and MSC are highlighted in red.

Fig. 3 illustrates the effects of combining the two correlation measures, normalized cross correlation (NCC) and multiscale correlation (MSC), on a sample 2-D image section of an NMJ, whereas Fig. 4 shows that combining both NCC and MSC spot detection algorithms detects faint spots while suppressing artefacts arising from the background. The NCC measure enables the accurate detection of the individual spots although they may be clustered together. In Fig. 5 we show that the proposed spot detection method is robust in distinguishing wild type from Bchs overexpressing ('EP+') for threshold values of *T*_1 _and *T*_2 _ranging from 0.3–0.7 and 15–30 respectively. A relatively small standard deviation of ~2% is observed for the classification accuracy.

### R-SVM Classification Results

After detection of spots by NCC and MSC, size, contrast and spot density feature measurements were extracted from the detected spots of a given NMJ image section (see Methods, Extraction of size, density and contrast). A stack of images was captured for every NMJ in every class, but the number of images in each stack varied depending on sample thickness. Each 2-D image section of an NMJ constitutes a data point that is represented by a feature vector with a total of 25 entries from which contrast, size and spot density measurements have 14, 10 and 1 entry respectively. Table 2 summarizes the number of NMJs and the corresponding data points used for comparing each pair of classes.

**Table 2:** Number of NMJs and data points (image sections) used to characterize differences between (i) wild type vs. EP+, (ii) wild type vs. *bchs1 *and (iii) wild type vs. bchs58

Class 1			Class 2		
Genotype	No. of NMJ	Data points	Genotype	No. of NMJ	Data points
Wild type	13	135	EP+	19	176
Wild type	21	217	*bchs*1	25	83
Wild type	21	217	*bchs*58	20	79

A radial basis function Support Vector Machine (R-SVM) classifier [24] was devised, into which the feature measurements were fed (see Methods, SVM classification). The R-SVM classifier quantified the differences in endolysosomal phenotypes between the wild type and each of the other three mutant genotypes. The R-SVM achieved good classification performance on previously unseen data by finding decision boundaries that optimally separate the wild type data points from those of the other genotypes. The classification scheme is expressed as three two-class problems i.e. wild type vs. *bchs1*, wild type vs. *bchs58 *and wild type vs. EP+.

The R-SVM was trained with data sets for each parameter from pairs of genotypes (i.e. *bchs*1 vs. WT or *bchs*58 vs. WT), and then challenged to classify images of unknown origin. Optimal classification accuracies of 78.1%, 72.2% and 82.0% were obtained for wild type vs. *bchs*1, *bchs*58 and EP+, respectively. Their corresponding SVM parameters representing the standard deviation of the Gaussian kernel *σ *and the penalty factor *φ *were as follows: (4, 1000), (40, 1000) and (8, 10) respectively for the three genotypes. Table 3 shows the confusion matrices for the three pairs. This is a significant achievement since, by simply analyzing an arbitrary 2-D section of an NMJ, we can accurately predict 78.1%, 72.2% and 82.0% of the time if the NMJ is a wild type or conversely a *bchs1*, *bchs58 *and EP+ mutant class respectively.

**Table 3:** Confusion matrices of spin wild type vs. (a). bchs1, (b). bchs58 and (c). EP+

		**Predicted Class**				**Predicted Class**	
		Wild type	*bchs*1			Wild type	*bchs58*
			
**Actual Class**	Wild type	**77.0**	23.0	**Actual Class**	Wild type	**71.9**	28.1
	*bchs*1	27.3	**72.7**		*bchs*58	27.0	**73.0**
	
		(a)				(b)	
				
		**Predicted Class**					
		Wild type	EP+				
					
**Actual Class**	Wild type	**78.5**	21.5				
	EP+	20.5	**79.5**				
				
		(c)					

**Figure 6:**
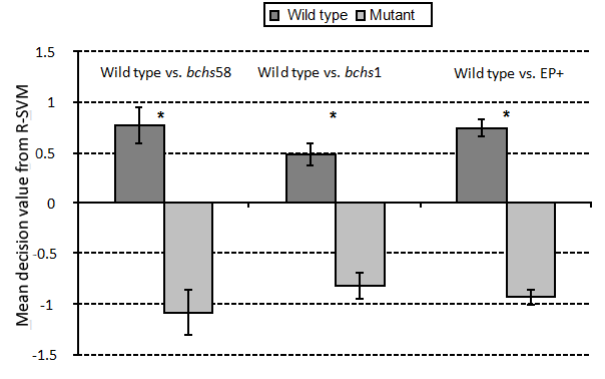
**Mean decision values for R-SVM classifications of samples**. Mean decision values obtained by the R-SVM on samples of wild type (dark gray) vs. *bchs58*, *bchs1 *mutant and EP+ overexpressing classes, respectively (light gray). A larger decision value indicates greater distance from the decision boundary and thus greater reliability of the classification.

In order to assess the ability of the SVM to predict the genotypic origin of a given NMJ image, mean decision values (reflecting degree of reliability of the assignment of mutant vs. wild type) were calculated. The mean decision values for all the individual decision values obtained by the SVM for each NMJ section of a particular category of mutant vs. wild type were averaged and plotted with standard errors (Fig. 6). The differences in the mean decision value between the wild type and mutant classes are statistically significant with a high confidence interval of 99.9%.

### Multivariate and Univariate Feature ranking

Using univariate and multivariate analyses, we assessed the value of various features to the SVM in determining its ability to predict genotypic origin (i.e., feature ranking). The purpose of feature ranking is to determine which features have the greatest discriminating power, and can thus be used most efficiently to assign a class (neurodegenerative vs. WT) to a given sample. This analysis also enables us to visualize the differences within each wild type vs. mutant pair.

The different feature categories, including 10 different size bins, 14 contrast bins, and the single density measurement were taken as individual features (see Methods, *Extraction of size, density, and contrast*). The Hotelling t-test [27] (see eqn. 5), was used to rank each of the features via the multivariate analysis approach (see Methods, Statistical data anlysis). A Hotelling multivariate *T*^2 ^statistic was applied in a sequential backward elimination scheme [37] to rank the contrast (*C*_1,...,14_), size (*S*_1,...,10_) and density (*D*) features based on their discriminating power. We start with a complete set of features and sequentially remove those features that reduce the *T*^2 ^value the least. Thus the feature of least importance is removed first and the most important is removed last. In the event that two or more features have the same *T*^2 ^value, we remove the feature that corresponds to the lowest univariate *T*^2^. The use of *T*^2 ^statistics is desirable due to its strictly monotonic function where its value for a subset of features is always less than or equal to that of the full set. Fig. 6 shows the monotonic decrease in the *T*^2 ^value where, at every stage, the least important feature is sequentially removed for each wild type vs. mutant pair.

**Figure 7:**
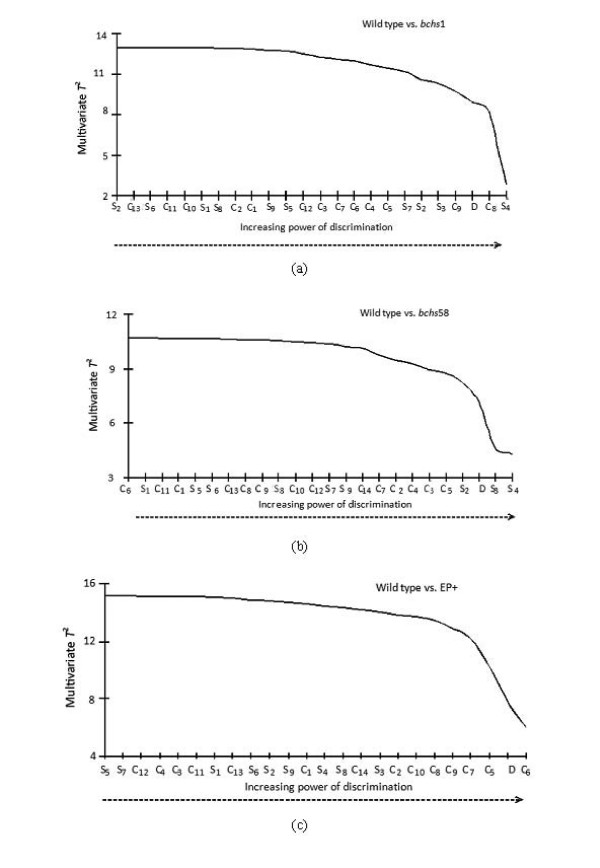
**Multivariate T2 ranking of features in increasing order of importance in determining the classification of phenotypes**. Effect of leaving out different contrast, size, and density (C, S, and D) feature classes on the ability of the R-SVM to discriminate between (a) Wild type vs. *bchs1 *(b) Wild type vs. *bchs58 *and (c) Wild type vs. EP+.

The feature describing spot density, *D*, appears as one of the top six most discriminating features for all three genotypes (Fig. 7). The smaller spots of sizes *S*_2_, *S*_3 _and *S*_4 _play an important role in discriminating wild type from *bchs*1 and *bchs*58 as shown in Fig. 7b, whereas spots of contrast levels *C*_5_, *C*_6_, etc. are crucial in discriminating wild type from EP+ as shown in Fig. 7c. Spots of mid level contrast *C*_8 _and *C*_9 _more effectively discriminate wild type from *bchs1 *whereas those of lower contrast *C*_3 _and *C*_5 _aid the discrimination of the *bchs58 *case.

### Visualizing the Differences in Genotypes – Univariate and Multivariate Analysis

**Figure 8:**
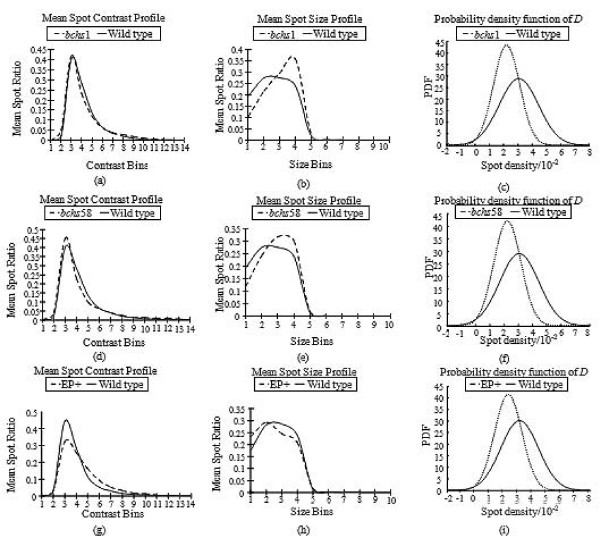
**Comparisons between histogram distributions of features in wild type vs. mutant classes (bchs1, bchs58 and EP+)**. (a), (d) and (g): mean contrast profiles, (b), (e) and (h): mean size profiles, and (c), (f) and (i): probability density functions of spots (endolysosomes) at NMJs of the given genotypes. Spot contrast is slightly increased for the Bchs overexpressor EP+ in comparison to wild type, whereas mean spot size is larger than wild type in the *bchs *mutants *bchs1 *and *bchs58*. Density is lower in all cases in comparison to wild type.

The differences between the wild type and mutant feature data were visualized by plotting on histograms the mean distribution profiles of endolysosomes from the different genotypes among the various contrast, size, and density bins (Fig. 8). In this way, the differences in the profiles of the mean spot size and contrast as well as variations in the spot density values could be compared visually between the wild type and the three mutant classes. A significant difference is observed in the mean spot size profiles (Fig. 8b, e) where the endolysosomal compartments are larger in the *bchs *mutants compared to the wild type. The mean contrast profiles of the *bchs *mutants in (Fig. 8a, d) are however very similar to the wild type. The converse is true for EP+ where the mean contrast profiles (Fig. 8g) show that, in general, the spots in EP+ have higher contrast levels than those in the wild type. Their mean spot size profiles, on the other hand, (Fig. 8h) are very similar.

Spot density is a scalar value and as such the differences in spot density values between the two groups are visualized from the corresponding probability density function (PDF) with a Gaussian distribution characterized by their respective mean and standard deviation in spot density. The spot density of the wild type endolysosomes is, in general, higher than those of any of the mutant classes (Fig. 8c, f, i). This is not an artifact of spot clustering in the mutants, since individual spots are resolvable from aggregations of spots. The filter profile was detectable within these more clustered regions.

**Figure 9:**
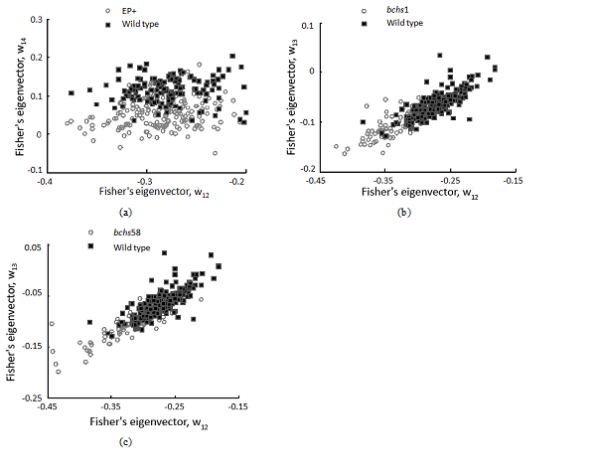
**Fisher's linear discriminant discriminates between wild type and mutant phenotypes**. 2-D scatter plots of wild type vs. EP+ (a) Wild type vs. *bchs1 *and (b) Wild type vs. *bchs*58 (c) in Fisher's space spanned by axes ω_12_, ω_13 _and ω_14_. Note that the scatter plots in (b) and (c) show that the wild type differs from *bchs1 *and *bchs58*, the two loss of function alleles, in the same way (spanned by the same projection axes and having similar distributions), but that WT deviation from the *EP+ *(Bchs overexpression) profile is clearly distinct from those of *bchs1 *and *bchs58 *(different projection axes and distributions).

Fisher's linear discriminant [26,27], shown in fig. 9, was used to compute the projection vectors **ω**_12_, **ω**_13 _and **ω**_14 _which optimally separate wild type vs. *bchs1*, wild type vs. *bchs58 *and wild type vs. EP+ respectively. These eigenvectors are then used to visualize the differences in distribution between the wild type and the mutant phenotypes. The discrimination between the wild type and mutant classes are visualized (Fig. 9b, c). As observed, wild type and EP+ were optimally discriminated along the axis ω_14 _(Fig. 9a) but axes ω_12 _and ω_13 _(not shown) do not contribute to this discrimination. Interestingly, the same two axes (ω_12 _and ω_13_) are both the best at discriminating wild type from mutants *bchs1 *and *bchs58 *(Fig. 9b, c). This implies that both *bchs1 *and *bchs58 *share common properties which set them apart from the wild type. This is consistent with these two genotypes both being strong loss of function alleles of *bchs*.

## Conclusion

We have developed a novel imaging-based method for quantifying neurodegeneration-associated changes in the endolysosomal compartment, an organelle whose function is strongly associated with the pathology of neurodegenerative diseases, in particular Alzheimer's disease[2]. This method may be useful as a potential tool for diagnosis and screening of the neurodegenerative pathology at the cellular level.

Image analysis was carried out on individual affected neurons in a Drosophila neurodegenerative model, *blue cheese*, a mutant which is thought to disrupt lysosomal function[11]. We were able to accurately detect and analyze GFP-labelled endolysosomes at the termini of motorneurons in situ, using novel automated segmentation and feature extraction algorithms. The key steps of the method are as follows: (1) A combination of normalized and standard cross correlation techniques is used to give accurate segmentation of compartments from image sections, even those with poor SNR; (2) Contrast, size and spot density measurements are extracted from individual endolysosomal compartments; (3) Impartial and automated diagnosis of the phenotypes via the R-SVM classifier is carried out, and (4) A backward elimination scheme is used to rank the various feature measurements in terms of their power to discriminate the mutant phenotypes from the wild type.

Fluorescently labelled endolysosomes in motorneuronal endings appear as spots with diverse contrast and intensity in 2-D image sections. Due to this variability, conventional image segmentation fails to detect these compartments. In contrast, our segmentation approach, which uses normalized cross-correlation and multi-scale correlation algorithms in succession, is sensitive yet selective to these compartments regardless of their contrast levels. Based on measurement of relatively few key features (spot density, intensity, and size) endolysosomes from neurodegenerative motorneurons can be accurately recognized and differentiated from the wild type. These differences are not obvious by inspection, and manual collection of such quantitative data from a large number of image samples is prohibitively labour-intensive. As a solution to this problem, the automated algorithm detects differences between the neurodegenerative mutant compartments and normal compartments with a highly statistically significant confidence interval of 99.9%.

The ranking of the different feature measurements against each other showed that the relative importance of a particular feature in distinguishing the origin of a sample varied between genotypes. For example, the differences between the wild type and phenotypes resulting from overexpression of the Bchs protein (EP+) are largely attributable to contrast variations. On the other hand, both size and contrast variations differentiated the wild type from either of the loss of function alleles *bchs1 *and *bchs58*. Density measurements were different in all three *bchs *genotypes (loss of function as well as overexpression) from the wild type. Ranking of feature importance makes it in principle possible to select the features most relevant to an assessment of the neurodegenerative phenotype, and increase the speed and accuracy of a diagnostic readout.

While the contrast, size, and density feature differences between genotypes are quite subtle, as is evident from Fig. 2, clearly the R-SVM classifier was able to distinguish the neurodegenerative (mutant) case from wild type, in all three genetic classes. Surprisingly, the application of the feature detection algorithm to the fluorescence images in some cases yielded results that were counter-intuitive to observations made by eye: strikingly, images of NMJs from neurons overexpressing Bchs appeared in general to have larger compartments, whereas the algorithm revealed that the compartments were actually of similar or even smaller size, but higher contrast.

Interestingly, both the neurodegenerative *bchs *loss of function alleles gave endolysosomal spot size profiles that were bigger than wild type, and this feature was prominent in determining genotypic origin. This may be related to the observation of enlarged lysosomal compartments in the neurodegenerative lysosomal storage diseases [38,39] and in the human Chediak-Higashi syndrome, which results from mutation in a gene related to *bchs *[40]. Another decisive feature difference, the lower density of the compartments at the NMJ in all three degenerative genotypes, is also consistent with the inefficient axonal transport of endolysosomes from neuronal cell bodies toward the termini (where images were obtained) that was observed in these same genotypes (A. Lim and R. Kraut, manuscript under revision).

An important finding of this study was that Fisher's linear Discriminant categorized both loss of function alleles according to the same eigenvectors, even given the subtlety of the differences in features. This demonstrates the power of the method to identify essential feature information and classify phenotype ("degenerative" vs. "normal") based on relatively small data sets. Moreover, subclasses of phenotype (e.g. Bchs overexpressor vs. *bchs *mutant) can be defined, since particular eigenvectors and distributions along these vectors appear to be typical for certain genotypic classes, as is seen in Fig. 9.

In conclusion, as one of the key early pathological features of neurodegenerative disease, it is significant that the endolysosomal phenotype is now open to analysis by an automated, potentially high throughput process. The technique described here may be useful in rapid screening of drug candidates, since it can easily be applied to images in cell culture models of neurodegeneration. This type of analysis should also allow rapid, unbiased evaluation of degenerative changes in different in vivo models, which are undetectable by traditional manual methods, and may ultimately be useful as a means of reliably diagnosing a systemic cellular condition that affects the endolysosomal compartment and predisposes neurons to degeneration.

## Competing interests

The authors declare that they have no competing interests.

## Authors' contributions

SK developed and implemented the algorithms for image processing, feature extraction and feature classification. SK also carried out the statistical analysis and drafted the manuscript. YW was responsible for the genetics, preparation of larvae and the acquisition of images. VV assisted in the implementation of the algorithms and the acquisition of images. RK conceived of the study, participated in its design and coordination, and helped to draft the manuscript.
